# Perpendicular Magnetic Anisotropy in FeRh Thin Films with Coexisting Magnetic Phases

**DOI:** 10.1002/advs.202510686

**Published:** 2025-08-20

**Authors:** Mengting Zou, Yali Xie, Huali Yang, Gengfei Li, Xilai Bao, Yanlong Yin, Wan Li, Wei Li, Chenxu Liu, Huatao Jiang, Mengqi Qian, Ruoan Zou, Baomin Wang, Run‐Wei Li

**Affiliations:** ^1^ School of Physical Science and Technology Ningbo University Ningbo 315211 P. R. China; ^2^ Ningbo Institute of Materials Technology and Engineering Chinese Academy of Sciences Ningbo 315201 P. R. China; ^3^ Eastern Institute of Technology Ningbo 315200 P. R. China

**Keywords:** FeRh thin films, magnetic phase transition, perpendicular magnetic anisotropy, spintronics

## Abstract

The advantages of high integration density, energy efficiency, and enhanced stability make the realization of perpendicular magnetic anisotropy (PMA) in single‐layer films a crucial step toward the development of advanced spintronic devices. A theoretical study predicted that the magnetic easy axis of FeRh film would reorient from out‐of‐plane (OP) to in‐plane (IP) directions during the antiferromagnetic‐ferromagnetic (AF‐FM) phase transition. However, few studies have observed an OP magnetic anisotropy in FeRh films. In this work, a continuous reorientation of the magnetic easy axis from OP to IP directions in FeRh film during its AF‐FM phase transition is demonstrated. Additionally, the anisotropy transition temperature increases with the film thickness. According to the magnetic domain imaging, the nucleation and initial growth of the FM domains at the AF state are dominated by magnetocrystalline anisotropy, leading to an OP easy axis in the FeRh thin film. With increasing temperature, shape anisotropy progressively dominates the magnetic properties of FeRh film, shifting the magnetic easy axis from OP to IP orientations. These findings not only demonstrate a novel anisotropy transition behavior in FeRh films, but also successfully induce PMA in such thick single‐layer films, providing critical experimental insights for the application of FeRh in spintronic devices.

## Graphical abstract

This work reveals the evolution of perpendicular magnetic anisotropy in FeRh thin films during the antiferromagnetic‐ferromagnetic phase transition. Temperature‐dependent measurements and magnetic domain imaging demonstrate reorientation of magnetic moments from out‐of‐plane to in‐plane, driven by competition between magnetocrystalline anisotropy and shape anisotropy. These findings demonstrate the potential of FeRh in tunable spintronic applications.

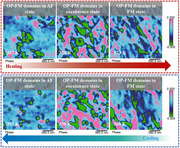

## 1 Introduction

As a crucial characteristic of magnetic materials, perpendicular magnetic anisotropy (PMA) significantly influences the progress of spintronic technologies. The development of next‐generation devices, such as magnetic tunnel junctions (MTJs) and magnetic random access memory (MRAM),^[^ ^1^, ^2^, ^3^ ^]^ requires a comprehensive understanding and flexible manipulation of PMA. Currently, several approaches, including stacking multilayer films,^[^ ^4^ ^]^ alloying rare earth transition metal,^[^ ^5^ ^]^ applying strain,^[^ ^6^, ^7^ ^]^ and selecting materials with intrinsic PMA,^[^ ^8^ ^]^ have been employed to induce PMA in diverse systems. However, these approaches also introduce additional challenges, impeding the development of PMA‐based spintronic devices. For example, stacking multilayer films involves complex and costly fabrication processes, and the presence of heterogeneous interfaces reduces stability and reliability of the devices. Alloying rare earth transition metal alloys is effective in attaining PMA, yet this approach is restricted by the high cost and complex magnetic characteristics of rare elements. Stress‐induced PMA is limited to specific material systems, and the stress relaxation associated with this method compromises stability and durability of the devices. Therefore, exploring novel materials with enhanced stability and developing approaches involving simplified manufacturing procedures to achieve reliable PMA are of great importance for advancing more efficient and miniaturized spintronic devices.^[^ ^9^ ^]^ Among the latest findings, the preparation of magnetic thin films on a MgO substrate ‐ renowned for its exceptional crystallinity and ability to induce robust PMA in certain magnetic materials ‐ has emerged as a particularly promising strategy for achieving reliable PMA.^[^ ^10^, ^11^, ^12^, ^13^, ^14^ ^]^ Recent studies have shown that in the CoFeB/MgO system, MgO significantly improves its thermal stability and plays a crucial role in achieving PMA.^[^ ^4^, ^15^ ^]^ However, the PMA in CoFeB/MgO system are primarily attributed to interface effect, which requires precise thickness control and is less favorable for stable operations in miniaturized devices. To address this challenge, K. Watanabe et al. developed CoFeB/MgO devices with columnar structures to stabilize PMA through shape anisotropy,^[^ ^16^ ^]^ thereby enabling robust PMA even in relatively thick films. Based on this, it can be concluded that achieving PMA in thicker single‐layer films exhibits great practical potential in developing advanced spintronic devices with improved stability, scalability, and cost‐effectiveness.

FeRh, which is a well‐known antiferromagnetic (AF) material at room temperature, undergoes a first‐order phase transition to a ferromagnetic (FM) state upon heating.^[^ ^17^ ^]^ Recent studies have highlighted the presence of PMA in FeRh films, despite Rh being a precious metal.^[^ ^18^, ^19^, ^20^, ^21^, ^22^ ^]^ Theoretical models suggested that both FeRh film thickness and interfacial interactions of FeRh/MgO significantly influenced the magnetic order and PMA.^[^ ^18^, ^19^ ^]^ Based on the theoretical models, Bordel et al. first experimentally observed PMA in a FeRh (150 nm)/MgO system at room temperature, which resulted from the interfacial stress.^[^ ^20^ ^]^ The MgO substrate enhances PMA in FeRh films through three key mechanisms: i) lattice‐matching‐induced (001) epitaxy with out‐of‐plane (OP) tensile strain, ii) intrinsic magnetocrystalline anisotropy of the FeRh(001) orientation, and iii) interfacial Fe 3d‐O 2p hybridization that may boost spin‐orbit coupling. These synergistic effects collectively optimize the PMA.^[^ ^21^, ^22^ ^]^ Additionally, subsequent work demonstrated that the Au/FeRh (10 nm)/MgO system also exhibited PMA at room temperature. The interfacial effects between Au and FeRh stabilized AF states at higher temperatures, leding to a discontinuous FM state during AF‐FM phase transition.^[^ ^23^ ^]^ Further theoretical research proposed a transition from intrinsic PMA to in‐plane (IP) magnetic anisotropy during the AF‐FM phase transition in FeRh/MgO films.^[^ ^24^ ^]^ However, FeRh/MgO films with thickness below 100 nm predominantly exhibit IP magnetic anisotropy, as the substantial shape anisotropy surpasses PMA resulting from interfacial effect and strain‐induced contributions.^[^ ^25^, ^26^, ^27^, ^28^ ^]^ In this study, we investigated the intrinsic PMA in FeRh/MgO films with FeRh thicknesses ranging from 30 to 80 nm during the AF‐FM phase transition. Our experimental results confirm that FeRh films exhibit PMA within the temperature range of 300–400 K. Notably, the temperature at which the magnetic anisotropy axis switches from OP to IP orientation increases with film thickness. This behavior is further supported by magnetic domain imaging, which provides insights into the underlying anisotropy mechanisms. The emergence of PMA is primarily associated with magnetocrystalline anisotropy, while its evolution during the phase transition is influenced by the competition between magnetocrystalline anisotropy and shape anisotropy. Our findings not only demonstrate the tunable and stable magnetic properties of FeRh thick films, but also provide critical insights into the analysis of PMA in magnetic phase transition systems, including those involving ferrimagnetic materials.

## 2 Results and Discussion

### 2.1 Crystal Structure of FeRh/MgO Films

**Figure 1:**
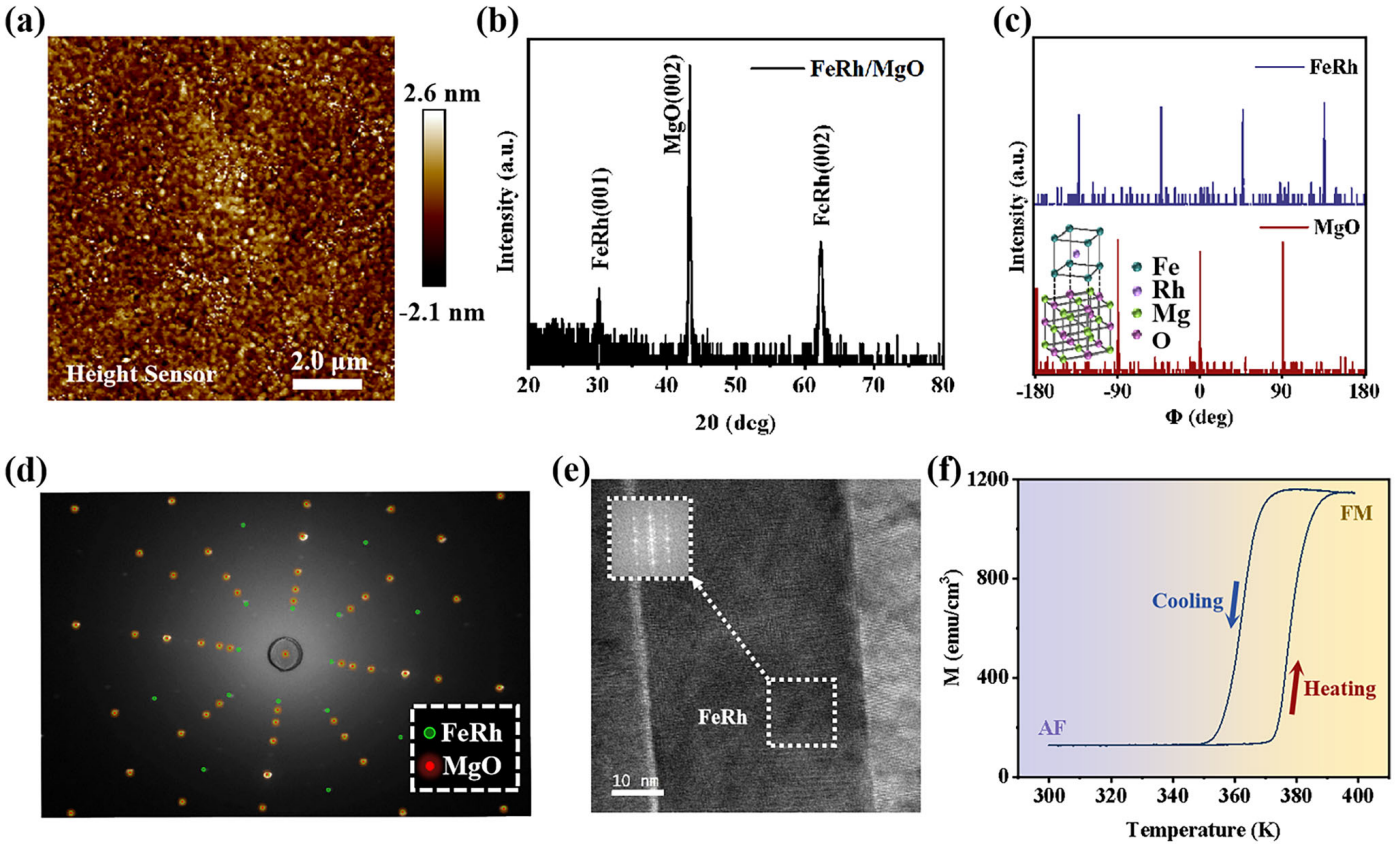
Crystal structure and magnetic phase transition of FeRh/MgO films. a) Scanning probe microscopy image of FeRh/MgO film. b) *θ*‐*2θ* and c) *Φ*‐scan curves of FeRh/MgO film measured through X‐ray diffraction. The inset in panel (c) is the schematic diagram of crystal structure which shows the epitaxial relationships of FeRh(001)/MgO(001). d) The Laue diffraction pattern of FeRh film. e) TEM image of FeRh/‐MgO film, the inset is the TEM diffraction pattern of the area marked with white dotted square. f) The magnetization‐temperature curve of the FeRh/MgO film measured under an in‐plane field of 2000 Oe.

**Figure** **1** shows the surface morphology and epitaxial quality of FeRh/MgO(001) thin films. The AFM image in Figure 1a illustrates a low surface roughness (Rq = 0.645 nm) over a scanned area of 10 × 10 µm. To confirm the epitaxial growth of the FeRh films, *2θ* scan and *Φ*‐scan curves were obtained, which are shown in Figure 1b,c, respectively. The results not only confirm the single‐crystalline state of the FeRh film but also indicate its epitaxial relationship with the MgO substrate. Specifically, with respect to MgO[100], FeRh[100] is rotated by 45° around the film normal.^[^ ^29^ ^]^ Further examination of the crystal quality was performed using X‐ray Back Laue Photography, as depicted in Figure 1d. The Laue patterns are round and clear without any signs of tailing or overlapping, indicating excellent single‐crystal quality of the FeRh films. The cross‐sectional TEM image shown in Figure 1e further confirms the single crystallinity of the FeRh film. The M‐T curve in Figure 1f shows a rapid and complete AF‐FM magnetic phase transition process, which proves the crystallinity of FeRh film through the magnetic measurements.

### 2.2 PMA Transition in Coexisting AF‐FM Phases of FeRh Thin Films with Varying Thicknesses

**Figure 2:**
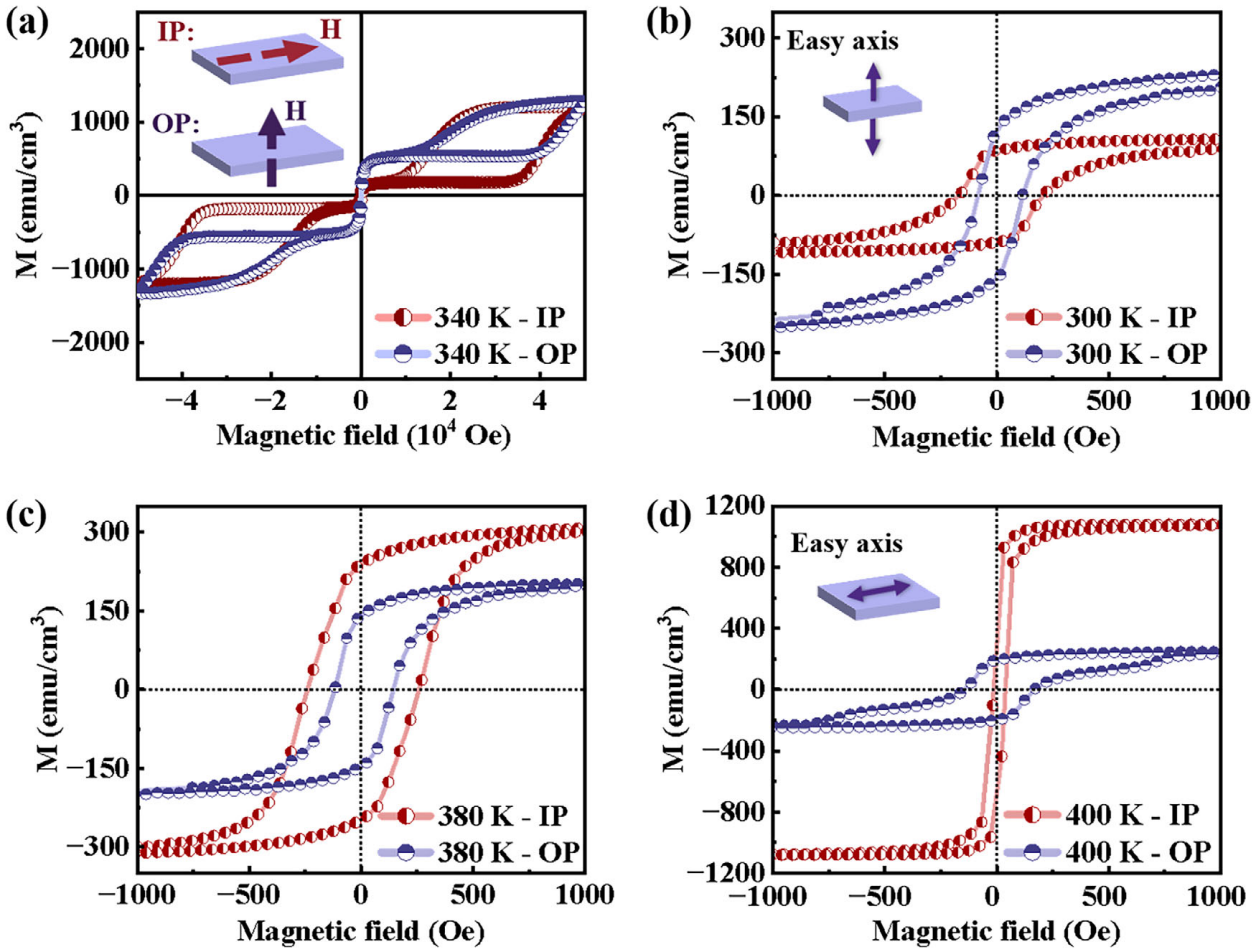
Hysteresis loops of FeRh films. a) M‐H loops of FeRh at 340 K (AF‐FM coexistence state) measured under magnetic fields of ±5 T along both IP and OP directions. b–d) M‐H loops measured at temperatures of 300, 380, and 400 K under an external magnetic field sweeping within ±1000 Oe. During the heating processes, no external magnetic field was applied. The insets in panel (a) are schematic diagrams of the M‐H hysteresis loops measured along the IP and OP directions, respectively. The insets in panels (b,d) illustrate the magnetic easy axis of the film at 300 and 400 K, respectively.

To explore the presence and evolution of PMA in the magnetic phase coexistence regime of FeRh thin films, a series of epitaxial FeRh films with thicknesses ranging from 30 to 80 nm were fabricated. For each film thickness, both IP and OP hysteresis loops were measured during the heating process with temperature increasing from 300 to 400 K. **Figure** **2** presents representative IP and OP hysteresis loops of the 40 nm FeRh film measured at various temperatures. In the first step, the temperature was adjusted to induce magnetic phase coexistence in the FeRh/MgO films, and a magnetic field of ±5 T was applied to achieve magnetic saturation. This saturation ensures accurate hysteresis loop measurements and facilitates the analysis of magnetic anisotropy in the FeRh films. The M‐H loops at 340 K, for example, are shown in Figure 2a. Under large magnetic field, the existence of a typical hysteresis loop is due to the phase transition induced by magnetic field. To remove the influence of field‐induced phase transitions, our analysis focuses on the magnetic response under low magnetic fields where temperature‐induced phase transition predominates. Therefore, we show a partial enlarged view of the hysteresis loops under magnetic field within ±1000 Oe at 300 (Figure 2b), 380 (Figure 2c), and 400 K (Figure 2d). At 300 K where the FeRh film is at its AF state, higher saturation magnetization and smaller coercive field are obtained under an OP magnetic field (Figure 2b), which indicates an observable PMA signal due to residual FM phases present in the FeRh film. The origin of the residual FM phase in FeRh films may be related to various factors such as electronic structure,^[^ ^30^ ^]^ phase transition dynamics,^[^ ^31^ ^]^ interfacial effects,^[^ ^32^ ^]^ and defects.^[^ ^33^ ^]^ The residual FM phase is magnetically active but distinct from the bulk properties, as evidenced by the significantly higher magnetization (≈1200 emu cm^−^^3^) in the bulk FM phase (Figure 1f). As the temperature rises to 380 K, the FeRh film exhibits AF‐FM phase coexistence, with the FM phases dominate. The M‐H curves indicate the competition between OP and IP magnetic anisotropy, as shown in Figure 2c. When a fully FM state is achieved in FeRh film at 400 K, the IP magnetization reaches ≈1100 emu cm^−^^3^ and the coercive field is extremely small, which indicates a strong IP magnetic anisotropy (Figure 2d). It can be concluded from above results that during the AF‐FM transition, the magnetic easy axis changes from OP to IP orientation. To further quantify the evolution of magnetic anisotropy in FeRh films, we extracted the temperature‐dependent magnetic anisotropy constant (K_U_) by comparing the IP and OP magnetization hysteresis loops measured at different temperatures. The calculation was based on the following approach: first, the IP anisotropy constant $K_{U}^{\mathrm{IP}}$ was estimated using the following expression:^[^ ^34^ ^]^

(1) $$K_{U}^{\mathrm{IP}}=\mu_{0}\int_{0}^{M_{S}}H_{x}dM_{x}-\mu_{0}\int_{0}^{M_{S}}H_{y}dM_{y}$$

Similarly, the magnetic anisotropy constant along the OP direction could be evaluated based on the following equation:

(2) $$K_{U}^{\mathrm{OP}}=\mu_{0}\int_{0}^{M_{S}}H_{x}dM_{x}-\mu_{0}\int_{0}^{M_{S}}H_{z}dM_{z}$$

Here, μ₀ denotes the vacuum permeability, and M_S_ represents the saturation magnetization. H*_x_*, H*_y_*, H*_z_*, M*_x_*, M*_y_*, and M*_z_* correspond to the applied magnetic fields and initial magnetization along the *x*, *y*, and *z* directions, respectively.

Accordingly, the magnetic anisotropy constant K_U_ at a given temperature can be calculated using the following expression:

(3) $$K_{U}{=K}_{U}^{\mathrm{IP}}{-K}_{U}^{\mathrm{OP}}$$

**Figure 3:**
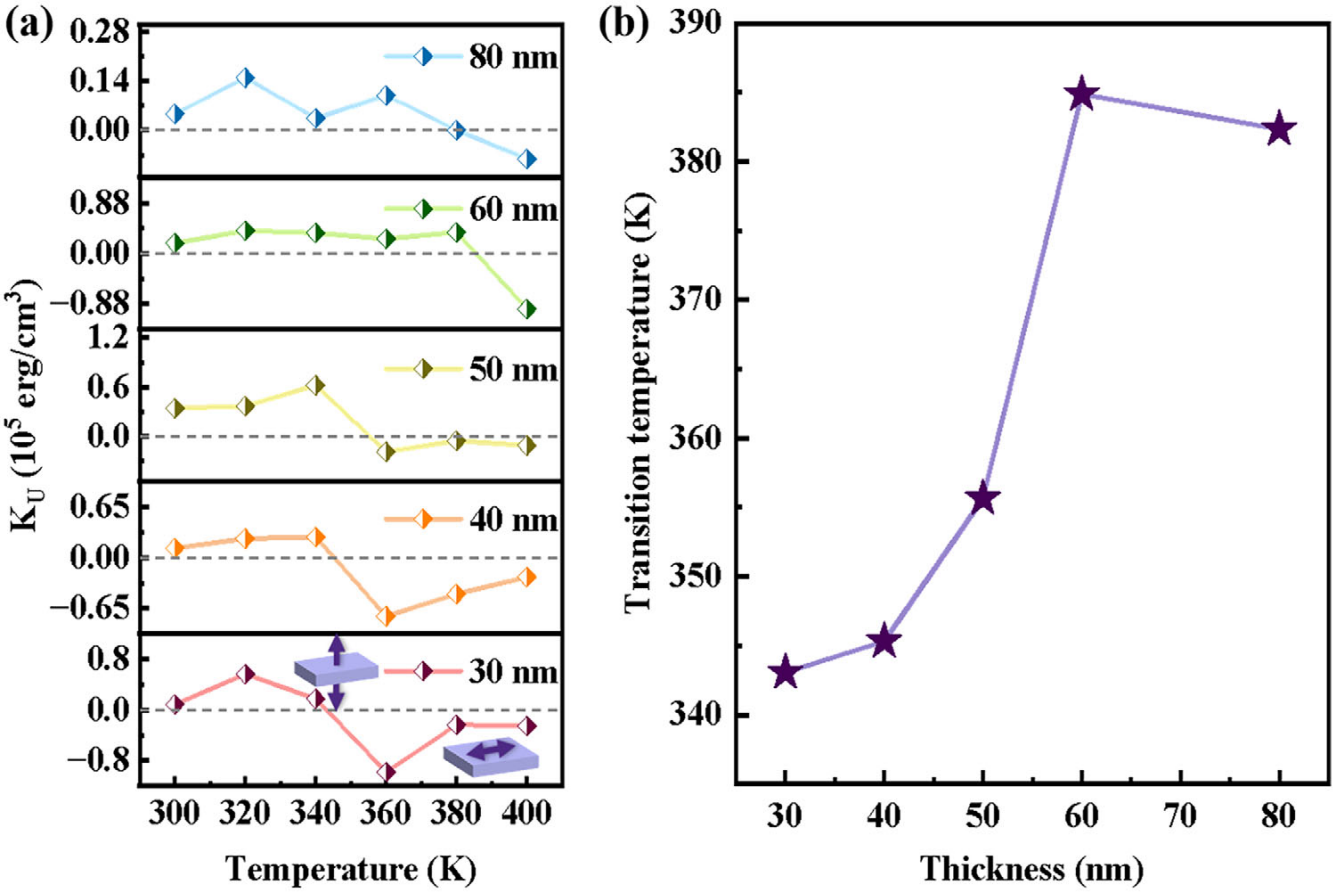
a) The temperature dependence of K_U_. b) The thickness dependence of magnetic anisotropy transition temperature.

Using the above calculation method, the magnetic anisotropy of a series of epitaxial FeRh films with different thicknesses (30, 40, 50, 60, and 80 nm) were systematically characterized. Based on the measured M‐H hysteresis loops, the temperature‐dependent magnetic anisotropy constants K_U_ were quantitatively extracted, as summarized in **Figure** **3**a. The results reveal a consistent trend for all samples with different thickness: with increasing temperature, the magnetic easy axis of FeRh films rotates from PMA toward IP orientation. At the beginning of the AF‐FM phase transition, PMA is initially enhanced. During the transition processes, the PMA weakens upon increasing temperature. As the transition is close to an end, the magnetic easy axis eventually rotates from OP to IP orientation. This quantitative analysis elucidates the dynamic evolution of magnetic anisotropy during the AF‐FM transition and highlights its strong dependence on film thickness.

To further investigate the thickness effect, the transition temperature, defined as the temperature at which K_U_ = 0, was extracted for each film (Figure 3b). The results show a clear thickness dependence: as the film thickness increases from 30 to 60 nm, the transition temperature rises from ≈344 to 385 K. This thickness‐dependent transition temperature may be associated with strain relaxation effects. As film thickness increases to 60 nm, partial relaxation of the interfacial strain may enhance the stability of the AF‐FM coexistence phases. The enhanced stability of AF‐FM coexistence phases requires higher temperatures to induce IP anisotropy in FeRh films. Notably, the transition temperature of the 80 nm film is slightly lower than that of the 60 nm film. The slight decrease in transition temperature at 60 nm compared to 80 nm suggests non‐monotonic strain relaxation, possibly due to variations in defect density or strain gradients during film growth, which could locally alter the energy landscape of the phase transition. It is worth noting that our FeRh films exhibit intrinsic PMA compared to multilayer systems, offering a key advantage by overcoming the limitations of ultrathin films, such as thermal instability and complex fabrication processes.^[^ ^16^ ^]^

This systematic evolution of magnetic anisotropy with thickness raises a fundamental question: What drives the reorientation of the easy axis during the AF‐FM phase transition in FeRh? We propose that the observed anisotropy transition stems from the competition between two energy terms: i) magnetocrystalline anisotropy, which favors OP magnetization in the strained FeRh(001) lattice, and ii) shape anisotropy, which promotes IP alignment due to the high saturation magnetization of FeRh thin films.

Fundamentally, for B2‐ordered FeRh, its magnetocrystalline anisotropy energy (E_MCA_) can be estimated using the following expression:^[^ ^34^ ^]^

(4) $$E_{\mathrm{MCA}}=K_{1}\left(\alpha_{1}^{2}\alpha_{2}^{2}+\alpha_{2}^{2}\alpha_{3}^{2}+\alpha_{3}^{2}\alpha_{1}^{2}\right)$$

Here, α_i_ represents the directional cosines between the magnetization direction and the crystal axes, while K_1_ denotes the magnetocrystalline anisotropy constant.

The shape anisotropy constant (K_Shape_) can be estimated by:

(5) $$K_{\mathrm{Shape}}=-\frac{1}{2}\mu_{0}M_{S}^{2}$$

where M_S_ is the saturation magnetization and μ_0_ = 4π × 10^−7^ H m^−1^.

At the AF state, K_1_ is ≈1–5 × 10^5^ erg cm^−3^. Taking into account the influence of residual FM ordering, its K_Shape_ is ≈−0.2 to −0.9 × 10^5^ erg cm^−3^. In this case, the effective anisotropy constant K_eff_ is calculated to be 0.1–4.8 × 10^5^ erg cm^−3^. Since K_eff_ > 0, FeRh films exhibits PMA characteristic. When FeRh is at its fully FM state, its K_1_ is ≈1 × 10^6^ erg cm^−3^, while its K_Shape_ is estimated to be −1 × 10^6^ erg cm^−3^. Consequently, the K_eff_ ≤ 0, indicating the easy axis of FeRh lies along IP direction.

### 2.3 Torque Curves of FeRh Films

To accurately and quantitatively investigate the change of magnetic easy axis in FeRh films during the phase transition, we employed the PPMS‐TQ option, a well‐established technique for assessing magnetic anisotropy, to measure the torque curves. The principle is as follows: the magnetic easy axis of a material corresponds to the direction in which its magnetic anisotropy energy is minimized. The anisotropy energy can be described by the equation:^[^ ^34^ ^]^

(6) $$E=K_{0}+K_{1}\times \sin2\theta +K_{2}\times \sin4\theta +\cdots$$

where K_0_, K_1_, K_2_, …, represent anisotropy constants that remain unchanged for a specific material at a given temperature. Generally, higher‐order terms like K_2_ are negligible, the portion of the anisotropy energy related to *θ* variation can be formulated as follows:

(7) $$E=K_{1}\times \sin2\theta$$

**Figure 4:**
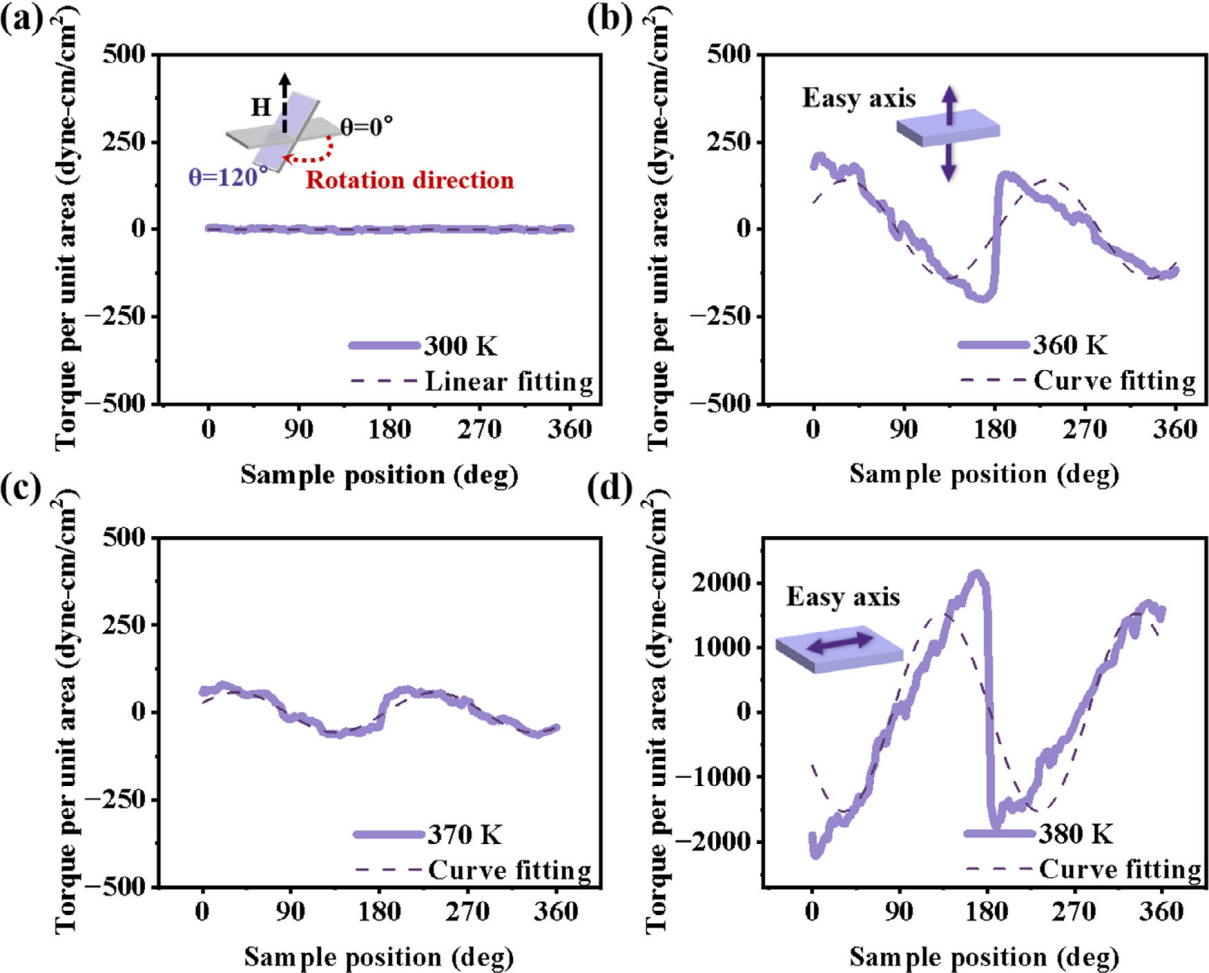
Measured torque and fitted curves of FeRh/MgO film. a–d) show the evolution of magnetic anisotropy in FeRh film at the temperature of 300, 360, 370, 380 K, respectively. The inset in panel (a) is schematic diagram of the testing angle and sample position. The insets in panels (b,d) show the magnetic easy axis of the film at 360 and 380 K, respectively.

When the energy of a system is related to an angle, the derivative of energy with respect to the angle, which is expressed as L = ±d_E_/d_θ_, is the torque (L). After analysis using standard testing procedures, the torque test has been established as L = d_E_/d_θ_ = K × sin*2θ*. Thus, by analyzing the torque curves, we can identify the magnetic easy axis of the sample by determining the direction in which the anisotropy energy is minimal. It should be noted that the angle between the film normal direction and the external magnetic field direction is defined as the sample position, i.e., *θ* (as shown in the inset of **Figure** **4**a). In Figure 4a, the torque value remains essentially constant throughout the rotation at 300 K, suggesting that the signal strength of the sample (AF‐FeRh) reaches the equipment test limit. As the temperature increases to 360 K, the FM phase gradually emerges. The torque curve, as shown in Figure 4b, can be well‐fitted by a sinusoidal function, and the fitted curve exhibits a period of π, which is a characteristic feature of uniaxial magnetic anisotropy. Obviously, when the torque curve L = 0 (θ = 0° and θ = 180°), the magnetic anisotropy energy reaches the minimum state, which means that the easy axis of the FeRh film at 360 K lies in OP direction. At 370 K (Figure 4c), as the FM phase grows further, the peak value of the TQ curve reduces, which indicates that the PMA weakens due to the increasing influence of shape anisotropy. When the temperature is further increased to 380 K (Figure 4d), a significant transformation of the torque curve shows that the easy axis of magnetization shifts to the IP direction.

### 2.4 Transport Measurements of FeRh Films

**Figure 5:**
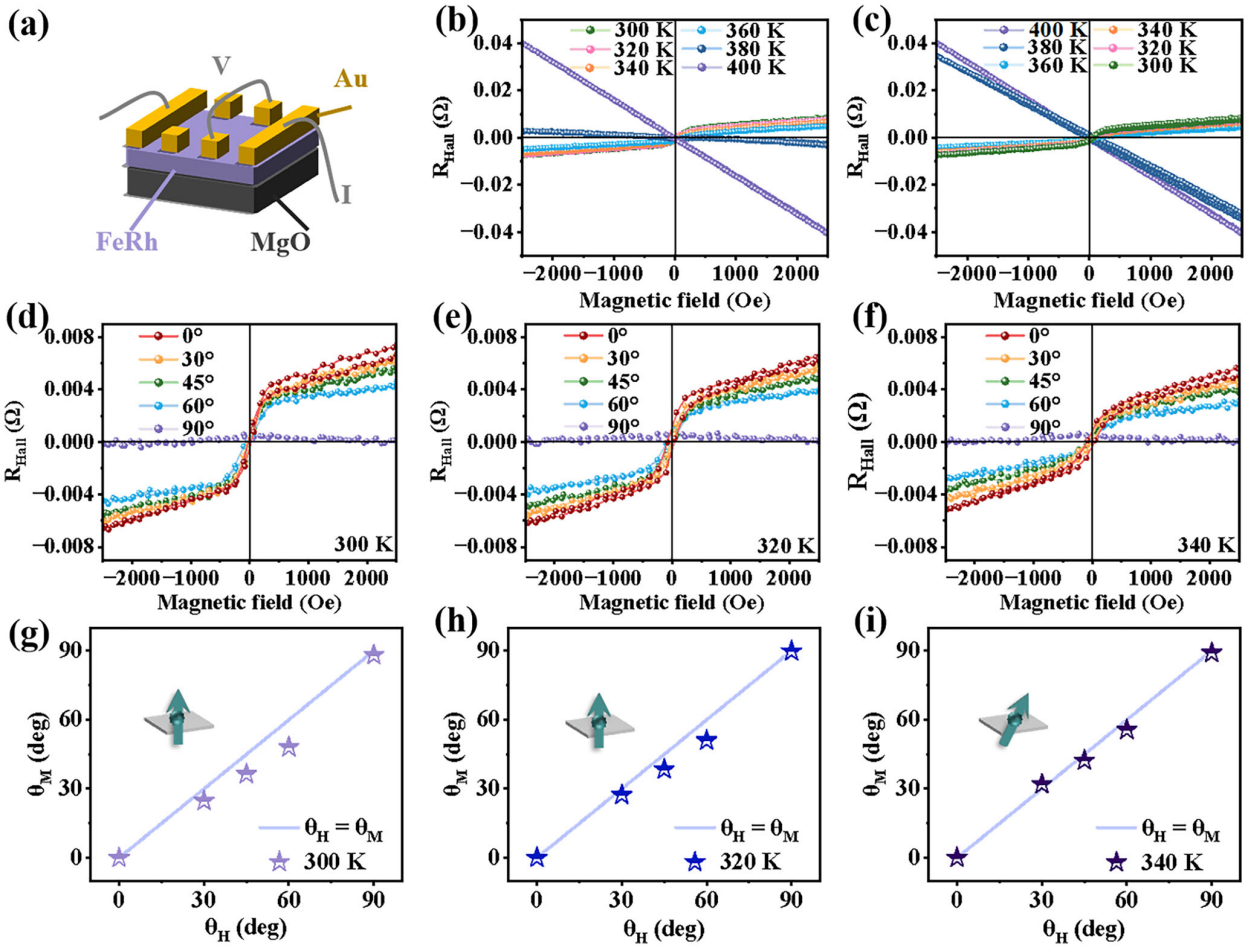
a) The schematic illustration of the FeRh film and the AHE measurement geometry. R_Hall_ curves with varying temperatures measured during heating b) and cooling c) process. Angle‐dependent Hall resistance curves of FeRh thin film at 300 d), 320 e), 340 K f). The relationship between θ_M_ and θ_H_ at 300 g), 320 h), and 340 K i) is calculated by curves of d–f). The insets in panels g–i) display the magnetic moment orientation.

At the initial stage of the phase transition from AF to FM, the ground state of FeRh is mainly composed of AF phases, while only sporadic FM regions are present. Consequently, the PMA signals are extremely weak within the temperature range of 300–350 K, and thus making it undetectable by the TQ option. To further investigate the magnetic anisotropy of FeRh films at relatively lower temperatures during its magnetic phase transition, we employed a high‐precision magneto‐transport technique to measure the R_Hall_‐H curves. A schematic diagram of the measured sample is shown in **Figure** **5**a. The R_Hall_‐H curves obtained during both heating and cooling processes are presented in Figure 5b,c. Due to the residue FM phase in FeRh, the Hall signal is appreciable at low temperature (300 K). As the temperature rises, the FeRh film undergoes a rapid phase transition from AF to FM states. During this process, there is a gradual transition of carriers in the transport properties. When the FM state is dominant, the properties of carriers tend to stabilize and the magnetic domains align more uniformly. Consequently, the anomalous Hall signal diminishes and the R_Hall_ exhibits a linear correlation with the external magnetic field.^[^ ^35^, ^36^ ^]^ These results imply that the employing angle‐dependent Hall measurements is necessary to gain deeper insights into the evolution of magnetic anisotropy within the low‐temperature regime.

Thus, we further measured the angular dependence of R_Hall_ at 300, 320, and 340 K, as illustrated in Figure 5d–f. Meanwhile, θ_H_, which is defined as the angle between the sample normal and the external magnetic field, θ_M_, which is defined as the angle between the sample normal and the magnetization direction, are utilized to analyze magnetic anisotropy.^[^ ^37^ ^]^ The angle θ_M_ can be quantitatively expressed as follows:

(8) $$\theta_{M}\left(\theta_{H}\right)=\arccos\left(\frac{R_{\mathrm{AHE}}^{S}\left(\theta_{H}\right)}{R_{\mathrm{AHE}}^{S}\left(\theta_{H}=0^{o}\right)}\right)$$

Then the magnetic anisotropy can be divided into three scenarios: i) The case of θ_H_ = θ_M_ indicates isotropical distribution of magnetic properties, which means that the direction of M consistently aligns with the direction of H. ii) The case of θ_H_ > θ_M_ indicates that the sample exhibits magnetic anisotropy and has an OP easy axis. iii) The case of θ_H_ < θ_M_ indicates that the sample exhibits magnetic anisotropy and has an IP easy axis. To investigate the correlation between θ_H_ and θ_M_, we plotted the θ_H_‐θ_M_ diagrams at temperatures of 300, 320, and 340 K, as shown in Figure 5g–i. It can be clearly concluded that the magnetic anisotropy of FeRh is oriented toward OP direction at 300 and 320 K. At 340 K, although the orientation of the magnetic easy axis becomes canted, the OP component still dominates the magnetic anisotropy.

### 2.5 Observation of Microscopic Magnetic Domain Structure in Phase‐Coexistence FeRh Films

Combined the R_Hall_ data with TQ curves and MPMS measurements, we conclude that PMA signals can be detected during the AF‐FM transition. This phenomenon may be related to the dominance of magnetocrystalline anisotropy. Specifically, in the early stages of the AF‐FM phase transition, the nucleation and growth of FM domains within the AF phase may not yet reach the critical size required to induce shape anisotropy. As the temperature increases, the expansion of the FM domains leads to a competition between magnetocrystalline and shape anisotropy. This competition could further result in a gradual tilt of the magnetic anisotropy direction, ultimately causing the easy axis to reorient from OP to IP direction.

**Figure 6:**
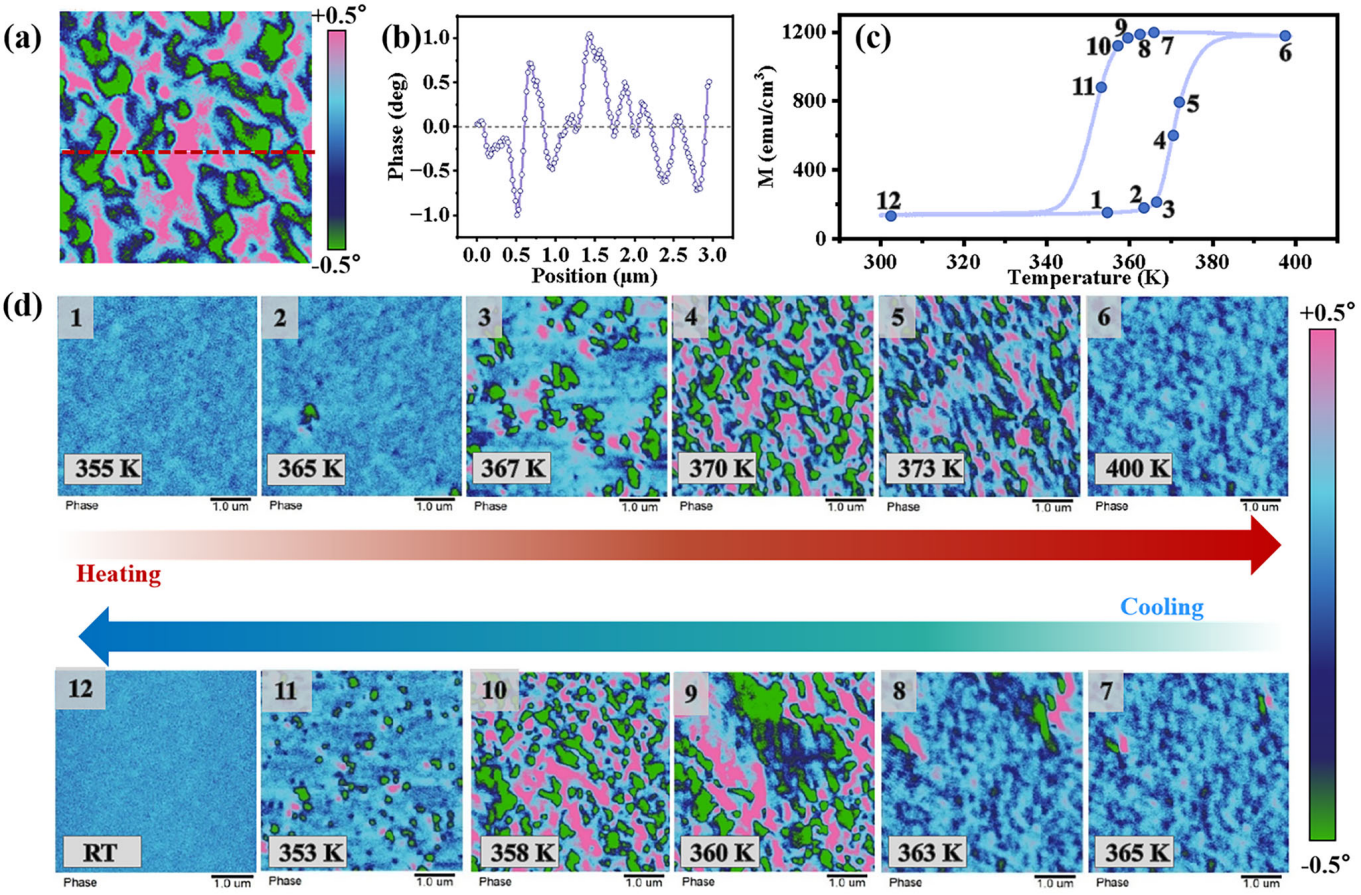
a) An example of MFM image. The MFM phase signal along the red dashed line in panel (a) is plotted in (b). c) The M‐T curve of the FeRh film used for MFM testing. d) In situ MFM images of the FeRh film at different temperatures, with the numbers in the upper‐left corner corresponding to those on the M‐T curve in panel (c).

To validate the previous hypothesis and gain a more intuitive understanding of the evolution of the magnetic domain structure in FeRh films during the AF‐FM phase transition, this study investigated the influence of the nucleation and growth of FM domains on magnetic anisotropy through magnetic domain imaging. A series of MFM images is shown in **Figure** **6**. It is important to note that the Phase in MFM images, manifested as the color intensity shown in the color bar, is proportional to the magnetic force component along the perpendicular magnetization direction of the sample (F_Magnetic_∝M^2^).^[^ ^38^ ^]^ In this study, to ensure a consistent comparison of MFM images at different temperatures, we fixed the phase of all images within an identical range of ±0.5°. For example, in Figure 6a, the bright pink and bright green regions correspond to magnetic domains with magnetic moments oriented upward and downward, respectively. For clarity, the phase signal along the red dashed line in Figure 6a is plotted as the curve in Figure 6b. It is evident that the positive phase signal corresponds to the bright pink region, indicating an upward magnetic moment, while the negative phase signal corresponds to the green region, indicating a downward magnetic moment.

After clarifying the principles of MFM measurements, we selected a typical FeRh film (its M‐T curve is shown in Figure 6c) for MFM analysis. This will facilitate the subsequent analysis of the evolution of FeRh magnetic domains during the AF‐FM phase transition process. It is important to note that the numbers in the upper‐left corner of the MFM images at different temperatures (Figure 6d) correspond to the numbers on the M‐T curve in Figure 6c. At ≈355 K (Figure 6d1), no bulk magnetic domains are observed due to the limitations of MFM resolution or the dominance of AF states. Notably, numerous scattered dark and light blue points suggest the presence of many nucleation points for the OP magnetic domains. Initially, bulk OP‐FM domains develop sporadically among the nucleation sites (Figure 6d2), followed by simultaneous growth and proliferation of these magnetic domains (Figure 6d3,4). As the temperature rises to 370 K, the size of a single domain is ≈245 × 570 nm. With further increase in temperature, the OP signal gradually weakens, and the size of a single domain reaches ≈100 × 900 nm at 373 K (Figure 6d5). This may indirectly support our hypothesis that as the temperature increases, the size of the FM domains increases, resulting in the gradual reorientation of the easy axis from OP to IP direction (Figure 6d6). During the cooling process (as shown in Figure 6d7–12), the overall trend in the evolution of magnetic domains during the cooling process contrasts with that observed during the heating stage. Although there are slight variations in the evolution of magnetic domains during the heating and cooling processes, these variations may be ascribed to the different ground states (AF or FM). The above results, on one hand, validate our hypothesis that the transition in FeRh magnetic anisotropy is attributed to the competition between magnetocrystalline anisotropy and shape anisotropy. On the other hand, these conclusions are consistent with previous findings from MPMS, TQ, and transport measurements, further corroborating the existence of PMA and its evolution during the phase transition process from a microscopic perspective.

## 3 Conclusion

In summary, we observed an intrinsic PMA in FeRh films at the early AF‐FM transition stage, which is closely related to the nucleation and growth of FM phases. As the temperature increases, the expansion of FM phases strengthens the PMA signal. However, continuous proliferation and growth of FM domains upon further heating weakens the PMA of FeRh films and ultimately shifting the OP magnetic easy axis toward IP direction. The transition of magnetic anisotropy in FeRh films is attributed to the competition between magnetocrystalline anisotropy and shape anisotropy. This conclusion also provides a clear explanation for the observed rise in the magnetic anisotropy transition temperature of thicker FeRh films. These insights pave the way for the development of FeRh‐based devices with switchable PMA, significantly expanding their applications in spintronics.

## 4 Experimental Section

FeRh films were deposited on (001)‐oriented MgO substrates. Prior to deposition, MgO substrates were preheated to 530 °C for 1 h in a vacuum chamber, and this temperature was maintained throughout the deposition process. A 40‐nm‐thick FeRh layer was deposited via DC sputtering, with a sputtering power of 100 W and an argon pressure of 4.5 mTorr. During the post‐deposition, FeRh films were annealed at 700 °C for 1.5 h under a base pressure below 1.0 × 10^−5^ Pa to ensure the formation of an ordered body‐centered cubic phase of FeRh. The surface morphology of the films was characterized using a Bruker Icon Atomic Force Microscope (AFM) in tapping mode. To confirm epitaxial growth, X‐ray Φ‐scans with a High‐Resolution X‐ray Diffractometer (HRXRD), X‐ray Back Laue Photography, and Transmission Electron Microscopy (TEM) were performed. The magnetic properties of the FeRh films were investigated using the Magnetic Properties Measurement System (MPMS), from which magnetization versus temperature (M‐T) and magnetization versus magnetic field (M‐H) curves were obtained. For a detailed analysis of the magnetic anisotropy, torque curves were measured at various temperatures using the torque magnetometer option of the Physical Property Measurement System (PPMS‐TQ). Additionally, transport measurements were carried out using the resistivity option of the PPMS. In order to microscopically understand the changes of PMA in FeRh, the temperature‐dependent magnetic domain structure of FeRh was further captured using magnetic force microscopy (MFM) mode on the Bruker Icon AFM.

## Conflict of Interest

The authors declare no conflict of interest.

## Data Availability

The data that support the findings of this study are available from the corresponding author upon reasonable request.
